# Predicted Disease-Specific Immune Infiltration Patterns Decode the Potential Mechanisms of Long Non-Coding RNAs in Primary Sjogren’s Syndrome

**DOI:** 10.3389/fimmu.2021.624614

**Published:** 2021-04-14

**Authors:** Caiqi Cheng, Jun Zhou, Ruiying Chen, Yo Shibata, Reina Tanaka, Jun Wang, Jiaming Zhang

**Affiliations:** ^1^ State Key Laboratory of Oral Diseases, National Clinical Research Center for Oral Diseases; Department of Orthodontics, West China Hospital of Stomatology, Sichuan University, Chengdu, China; ^2^ Department of Conservative Dentistry, Division of Biomaterials and Engineering, Showa University School of Dentistry, Tokyo, Japan; ^3^ School of Stomatology, Hospital of Stomatology, Tianjin Medical University, Tianjin, China; ^4^ Department of Oral and Maxillo-facial Implantology, Shanghai Ninth People’s Hospital, School of Medicine, Shanghai Jiaotong University, Shanghai, China; ^5^ National Clinical Research Center for Oral Diseases, Shanghai, China; ^6^ Shanghai Key Laboratory of Stomatology & Shanghai Research Institute of Stomatology, Shanghai, China; ^7^ Department of Orthopedics, Tongji Hospital, Tongji Medical College, Huazhong University of Science and Technology, Wuhan, China; ^8^ Cancer Center, Union Hospital, Tongji Medical College, Huazhong University of Science and Technology, Wuhan, China

**Keywords:** Sjogren’s syndrome, sicca, immune infiltration, long non-coding RNA, chemokine

## Abstract

Primary Sjogren’s syndrome (pSS) is a chronic progressive autoimmune disease with clinical phenotypic “Sicca symptoms”. In some cases, the diagnosis of pSS is delayed by 6–7 years due to the inefficient differential diagnosis of pSS and non-SS “Sicca”. This study aimed to investigate the difference between these two diseases, and in particular, their immunopathogenesis. Based on their gene expression profiles, we systematically defined for the first time the predicted disease-specific immune infiltration pattern of patients with pSS differentiated from normal donors and patients with non-SS “Sicca”. We found that it was characterized by the aberrant abundance and interaction of tissue-infiltrated immune cells, such as a notable shift in the subpopulation of six immune cells and the perturbed abundance of nine subpopulations, such as CD4+ memory, CD8+ T-cells and gamma delta T-cells. In addition, we identified essential genes, particularly long non-coding RNAs (lncRNAs), as the potential mechanisms linked to this predicted pattern reprogramming. Fourteen lncRNAs were identified as the potential regulators associated with the pSS-specific immune infiltration pattern in a synergistic manner, among which the *CTA-250D10.23* lncRNA was highly relevant to chemokine signaling pathways. In conclusion, aberrant predicted disease-specific immune infiltration patterns and relevant genes revealed the immunopathogenesis of pSS and provided some clues for the immunotherapy by targeting specific immune cells and genes.

## Highlights

(1) This study systematically and for the first time defined the predicted disease-specific immune infiltration patterns in primary Sjogren's syndrome, characterized by the altered abundance and interaction of tissue-infiltrating immune cells.

(2) Essential genes, particularly long non-coding RNAs, are associated with this aberrant reprogramming of predicted immune infiltration patterns.

(3) The lncRNA CTA-250D10.23 is a potential regulator associated with the chemokine signaling pathway and immune infiltration

## Introduction

Sjogren’s syndrome (SS) is a chronic progressive autoimmune disease presented with clinical phenotypic “Sicca symptoms” on mucosa surfaces (mouth and eyes dryness) and characterized by damage and dysfunction of exocrine glands and principally reduced secretory functions of the salivary and lacrimal glands (1, 2). In particular, SS has been reported to occur in isolation or in combination with another systemic autoimmune rheumatic disorder and, thus is subdivided into primary SS (pSS) and secondary SS (sSS). The latter is known to be accompanied by lesions due to immunologic abnormalities or vasculitic involvement, such as systemic lupus erythematosus (SLE), rheumatoid arthritis, systemic sclerosis, and dermatomyositis (2–4).

The term “Sicca syndrome” has been used as a synonym for SS (5). However, “Sicca syndrome” and SS are not equivalent, neither clinically nor pathologically. Patients complaining of oral and ocular dryness, typically termed as “Sicca syndrome”, but not fulfilling the criteria of pSS, are referred to as non-Sjögren’s syndrome sicca (non-SS) patients (6, 7). It has been reported that up to 30% of people older than 65 y of age might exhibit dryness of both eyes and mouth (8), whereas the prevalence of pSS is known to be 0.03 to 2.7% depending on the applied diagnostic criteria (9). In some cases, the diagnosis of patients with pSS is delayed by 6–7 years after the onset of the disease due to the inefficient differential diagnosis of pSS and non-SS. Therefore, it is worthwhile to investigate the difference between these two groups of patients, which would be beneficial to the early diagnosis of pSS.

The development of pSS has been reported to be influenced by the interaction between multiple environmental factors and individual genetic susceptibility. From the immunopathological perspective, autoimmune epithelitis in pSS patients is a widely supported mechanism suggesting the involvement of inflammatory lesions of the epithelium with immune responses to the autoantigens Ro/SSA and La/SSB (10, 11). The consequence of abnormal interaction cycle between the epithelial cells of the salivary gland (SGECs) and immune cells results into the establishment of long-term autoimmune responses (10–12). Despite the accumulated knowledge on the epithelial–immune interaction cycle revealing the roles of immune cells in pSS, the systematic pattern of immune infiltration and cell interaction, which might provide a global perspective and a comprehensive image of the epithelial–immune interaction cycle, has not been defined yet.

To date, topically and systemically administered symptomatic treatments are considered as the primary therapies for the management of both diseases (8). Due to lack of understanding of the mechanisms of pSS, effective immunotherapy is still missing. Therefore, identification and comparison of the molecular mechanisms of these two diseases could identify disease-specific targets and facilitate the development of novel immunotherapy approaches for the treatment of pSS. In this context, we employed an integrated bioinformatics method to define the patterns of predicted immune cell infiltration in pSS and “Sicca” and then identified the disease-specific features of pSS by comparing these two diseases. Based on these defined features, we built the links between the common or disease-specific immune features and transcriptomes in each case, serving as a reference to advance the early diagnosis and treatment of pSS.

## Materials and Methods

### Data Preprocessing and Differential Expression Analysis

The GEO dataset: GSE40611 was obtained from the National Center For Biotechnology Information (NCBI) Gene Expression Omnibus (GEO) (7) using the “Sjogren’s Syndrome” and “parotid” criteria. This microarray dataset has been based on the platform of Affymetrix Human Genome U133 Plus 2.0 Array (HG-U133_Plus_2) and contains parotid tissue samples from 20 healthy donors, 19 patients with Sjogren syndrome, and 20 patients with “Sicca syndrome”. Before differential expression analysis, background correction and quantile normalization were performed using the robust multi-array analysis (*RMA*) method with the *limma* R package (v3.11) (13), thus generating the normalized gene expression matrix. In addition, differentially expressed genes (DEGs) were identified using the criteria of false discovery rate (*FDR*) <0.05 or <0.1, depending on the proportion of DEGs among the total.

### Definition of Predicted Immune Infiltration Patterns

The relative abundance of 22 types of tissue infiltrating immune cells, including CD8+ T-cells, naïve CD4+ T-cells, resting memory CD4+ T-cells, activated memory CD4+ T-cells, follicular helper T-cells, regulatory T-cells (Tregs), gamma delta T-cells, three types of macrophages (M0, M1, and M2), naïve B-cells, memory B-cells, plasma cells, resting natural killer (NK) cells, activated NK cells, monocytes, resting dendritic cells, activated dendritic cells, resting mast cells, activated mast cells, eosinophils, and neutrophils were estimated using the *CIBERSORT* deconvolution algorithm (CIBERSORT R script v1.03) (14) based on the *LM22 Signature* (https://cibersort.stanford.edu/, **Supplemental Table 1**). Then, the normalized gene expression matrix was converted to the immune cell matrix, which was further filtered according to the criteria of P <0.05. Comparisons of the relative abundance in pSS or “Sicca” with those in control (healthy donors) were performed using the *Wilcoxon signed-rank test*. Principal component analysis (PCA) was also performed to determine the overall difference between the three groups. The associated networks of predicted 22 types of tissue infiltrating immune cells were constructed using Pearson correlation analysis. The immune states were defined by three indexes, including the overall immune score, immunostimulator score, and immunoinhibitor score, which were estimated using the ESTIMATE and ssGSEA algorithms (**Supplemental Table 2**) (15, 16).

### Identification of Gene Modules Associated With The Predicted Disease-Specific Immune Infiltration Patterns

Considering the variance of gene expression, the top 25% including 5,836 genes were inputted in weighted correlation network analysis (*WGCNA*) to build the gene network using the *WGCNA* R package (v1.69) (17). Briefly, the soft-thresholding power was determined using the *softConnectivity* and *pickSoftThreshold* functions. When the scale-free topology fit index reached 0.9 at powers <30 and the network harbored an appropriate network connectivity, the soft-thresholding power was considered suitable for constructing a scale-free network. Then, the adjacency matrix was calculated under this power and converted into the topological overlap matrix (TOM), based on which gene modules were identified using the *minModuleSize* = 30 and *deepsplit* = 2 parameters in the *Dynamic Tree Cut algorithm* function and further merged using the *hierarchical clustering* and *merged dynamic algorithm* (*cutheight* = 0.25). Finally, the eigengenes representing the corresponding modules were employed to calculate the correlation between the modules and features, and quantified using the *Pearson correlation*. Modules with a *Pearson correlation coefficient* r >0.4 and P <0.05 were considered as significant modules.

### Functional Annotations of Immune Infiltration-Relevant Gene Modules

The *ClusterProfiler* R package (v3.14.3) was employed to analyze the Gene Ontology (GO) and Kyoto Encyclopedia of Genes and Genomes (KEGG) to annotate immune infiltration-relevant genes (18). Based on hypergeometric distribution, the *enrichGO* and *enrichKEGG* functions were utilized to perform enrichment analysis for GO terms and KEGG pathways, the results of which were visualized with bubble plots. Gene Set Enrichment Analysis (GSEA) and single-sample GSEA (ssGSEA) were employed to determine whether an *a priori* defined set of genes showed statistically significant differences between the groups or samples using the GSEA software v4.1.0 (19) and *GSVA* R package (v3.11) (16).

### Identification of Essential Genes Associated With the Predicted Common or Disease-Specific Immune Infiltration Patterns

The critical genes contributing to the common features of the immune infiltration patterns of pSS and “Sicca” were defined as the common DEGs correlating with the abundance of the commonly infiltrated immune cells. The correlations between the common DEGs and immune cell abundance were estimated using the *Pearson correlation coefficient*. The genes associated with predicted disease-specific immune infiltration patterns were defined as the gene modules that were specifically and statistically significant for pSS or “Sicca” in *WGCNA*. In addition, essential lncRNAs contributing to immune infiltration patterns were identified using the annotations of the *Ensembl* database (Release 98).

### Independent Dataset Validation

Independent GEO dataset: GSE23117 was employed to validate the expression of lncRNAs in pSS patients. With the same platform with GSE40611, GSE23117 contains 15 gene expression profiles of minor salivary gland of pSS patients and control (20). Data process and method of dataset validation were the same with GSE40611.

### Statistical Analysis

Comparisons between the two groups were performed using the unpaired two-tailed Student’s *t*-test, whereas the one-way ANOVA statistics method was employed for comparison among multiple groups. All data were displayed as means ± SD and analyzed using the GraphPad Prism software version 7.0. P < 0.05 was considered statistically significant.

## Results

### Disease-Specific Predicted Immune Infiltration Pattern and Enhanced Immune Response in pSS

To investigate the functions and mechanisms of immune infiltration in pSS, we first investigated the predicted immune infiltration pattern using an estimation based on the microarray data. Given that the main common manifestations of pSS and “Sicca” are similar, we speculated that the comparison of the immune infiltration pattern of pSS with that of “Sicca” would be disease-specific, thus revealing the mechanisms underlying the development of pSS.

**Figure 1:**
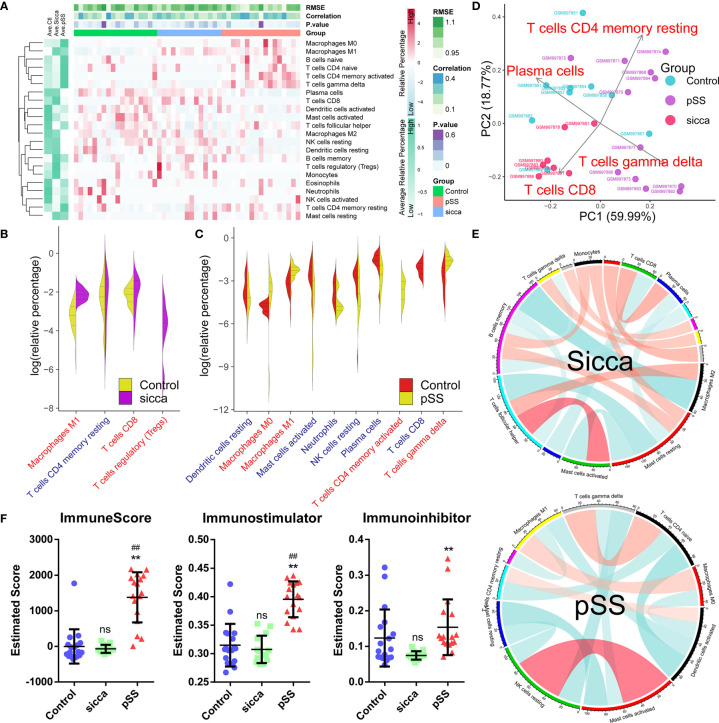
Predicted disease-specific immune infiltration patterns of pSS and “Sicca”. **(A)** The heatmap of the relative number of 22 types of immune cells estimated using the CIBERSORT algorithm and hierarchical clustering shows the landscape of the infiltration characteristics of pSS, “Sicca”, and control. RMSE, root mean squared error; correlation, Pearson correlation coefficient; P value; pSS, primary Sjögren syndrome. **(B)** Comparisons of infiltrating immune cell subpopulations of pSS *versus* control. Eleven subpopulations were identified as disease-relevant features of pSS. Blue, decreased subpopulation; red, increased subpopulation. **(C)** Comparisons of infiltrating immune cell subpopulations of “Sicca” *versus* control. Four subpopulations were identified as disease-relevant features of “Sicca”. **(D)** Principal component analysis (PCA) of 22 types of infiltrating immune cells. Four types of immune cells, including resting memory CD4 T-cells, CD8 T-cells, gamma delta T-cells, and plasma cells, were the principal elements contributing to the differences between the control, “Sicca”, and pSS. **(E)** Association network of infiltrating subpopulations estimated with the Pearson coefficient. Red, positive correlation; green, negative correlation. **(F)** Immune states of control, “Sicca”, and pSS estimated by ImmuneScore, Immunostimulator, and Immunoinhibitor using the ssGSEA algorithm. ##P < 0.01, pSS *versus* control; **P < 0.01, pSS *versus* “Sicca”; ns, not significant.

As shown in **Figure 1A** and **Supplemental Table 3**, we estimated the relative proportion of the 22 subpopulations of immune cells, defined as the “*immune infiltration pattern”*, using the *CIBORSORT* algorithm. We observed that the structure of predicted immune infiltration in pSS differed significantly from that in normal or “Sicca” and was characterized by a notable shift in the numbers of six subpopulations, including M0 macrophages, M1 macrophages, naïve B-cells, naïve CD4 T-cells, memory activated CD4 T-cells, and gamma delta T-cells, whereas the remaining subpopulations were decreased or did not differ in pSS compared with control or “Sicca”. Indeed, we found that the abundance of four subpopulations of cells was significantly increased (P *< 0.05, Wilcoxon signed-rank test*) in pSS *versus* normal samples, whereas it was decreased in six subpopulations. In contrast, only three subpopulations were shown to be increased, whereas one was decreased in “Sicca” compared with normal samples (**Figures 1B, C**, **Supplemental Table 4**). We identified that the increased number of M1 macrophages was the common feature in pSS and “Sicca”, suggesting that this feature might contribute to the common manifestations of these two diseases.

In the context of these differences in immune infiltration patterns, we aimed to explore whether these features were disease-specific, thus contributing to the distinguishment between these two diseases. As shown in **Figure 1D**, *principle component analysis* (PCA) revealed that four subpopulations, including memory activated CD4 T-cells, CD8 T-cells, gamma delta T-cells, and plasma cells, were identified as the main contributors; however, the boundary between pSS and “Sicca” was not distinct. Moreover, we noted that the obtained immune infiltration pattern failed to distinguish between the samples of “Sicca” and normal. We also investigated the association of subpopulations to explore disease-specific regulation patterns (**Figure 1E**). Accordingly we found that the diversity of association network was lower in pSS compared with “Sicca” and was characterized by a reduced connectivity of memory B-cells and a shift in the “leading subpopulation” defined as the cell type harboring the largest connectivity number in the association network. (pSS: activated dendritic cells, 4°; “Sicca”: memory B-cells, 6°)

As we identified differences in immune infiltration, we also investigated whether the overall status of the immune system differs among the three groups. Indeed, the immune scores of pSS estimated using ssGSEA, and in particular, the immune-stimulator score, were found to be significantly elevated, whereas no difference was observed between the “Sicca” and the normal groups (**Figure 1F**), suggesting a stronger immune response in pSS compared with Sicca and normal. Taken together, we demonstrated that the predicted disease-specific immune infiltration pattern of pSS was characterized by the sharped structure of infiltrated subpopulations and a reprogrammed regulation pattern, resulting in an elevated overall immune response compared with that in “Sicca” and normal.

### Common Genes and Long Non-Coding RNAs Associated With The Consistent Predicted Immune Infiltration Features of pSS and “Sicca”

Given that we found a common feature of immune infiltration in pSS and “Sicca”, namely the increased abundance of M1 macrophages, we explored the common mechanisms underlying these two diseases before investigating the disease-specific mechanisms of pSS.

**Figure 2:**
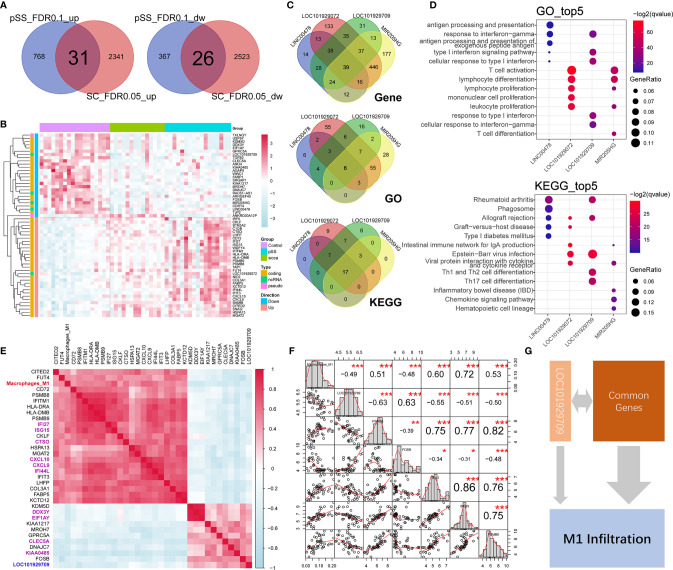
Common genes and lncRNAs associated with the consistent immune-infiltration features of “Sicca” and pSS. **(A)** Overlap of up-regulated or downregulated genes in “Sicca” and pSS compared to control. A total of 57 genes were identified as the common genes of the two diseases. **(B)** Heatmap of 57 common genes shows five lncRNAs, including *LINC00478*, *LOC101929072*, *LOC101929709*, *MIR205HG*, and *RAD51-AS1*. **(C)** Coexpressed genes and common GO/Kegg items relevant to *LINC00478*, *LOC101929072*, *LOC101929709*, and *MIR205HG*. **(D)** Top-five significant GO and Kegg items of *LINC00478*, *LOC101929072*, *LOC101929709*, and *MIR205HG*. **(E)** Correlation of M1 macrophage infiltration with common genes and lncRNAs. Heatmap shows the 32 common genes and 1 lncRNA, *LOC101929709* (blue) harboring a Pearson correlation coefficient larger than 0.4, among which the top-10 significant genes are labeled in purple color. **(F)** The M1 macrophage-relevant genes coexpressed with *LOC101929709* lncRNA. **(G)** A model of potential mechanisms of common genes and lncRNAs linked to M1 macrophage infiltration in “Sicca” and pSS. *P<0.05, **P<0.01, ***P<0.001.

As shown in **Figures 2A, B**, **Supplemental Figure 1**, **Supplemental Table 5**, and **Supplemental Table 6**, we identified a total of 57 commonly up-regulated or downregulated genes in pSS or “Sicca” *versus* normal, among which five long non-coding RNAs (lncRNAs), *LINC00478*, *LOC101929072*, *LOC101929709*, *MIR205HG*, and *RAD51-AS1* were shown to account for ~8% of the overlapped differentially expressed genes (DEGs). Due to the notable roles of lncRNAs elucidated in recent years, we speculated that these five lncRNAs might be relevant to the common features of pSS and “Sicca” and aimed to investigate their functions if shown to be significantly relevant.

To address this question, we calculated the *Pearson correlation coefficient* of genes and identified the coexpressed genes of lncRNAs (r >0.4 or <−0.4 and P *< 0.05*), including 144, 184, 245, 759, and 766 genes for *RAD51-AS1*, *LINC00478*, *LOC101929709*, *LOC101929072*, and *MIR205HG* lncRNA, respectively. The functional annotations of these relevant genes revealed that *LINC00478*, *LOC101929709*, *LOC101929072*, and *MIR205HG* were highly functionally relevant, sharing 39 common genes, six items of *GO* biological processes, and 17 *KEGG* pathways, whereas *RAD51-AS1* failed to be functionally annotated because of the biological irrelevance of its coexpressed genes (**Figure 2C**). Interestingly, we observed that these four lncRNAs were involved in multiple immune-related biological processes and signaling pathways, such as lymphocyte function, type I interferon signaling pathway and chemokine signaling pathway (**Figure 2D**), suggesting the potential synergistic effects of lncRNAs on immune regulation.

In the context of the high relevance of these lncRNAs, we aimed to explore whether they were associated with the predicted increased abundance of M1 macrophages in pSS and “Sicca” and the mechanisms underlying their connection to the other common genes. We calculated the *Pearson correlation coefficient* of the abundance of M1 macrophages and common genes. As shown in **Figure 2E**, we identified a total of 32 genes, accounting for ~56% of the total common genes, with high relevance to M1 infiltration. To investigate if these 32 genes were included in the M1 gene signature of LM22 prediction reference, we took their intersection with M1 gene signature to avoid the potential circular analysis. As shown in **Supplemental Figure 2**, only six out of 32 were included in the M1 gene signature. Moreover, we considered the top 10 genes ranked by the *Pearson correlation coefficient* as the key relevant genes in M1 infiltration, with only one lncRNA, *LOC101929709*, reaching statistical significance (*r = −0.49*, P *< 0.001*). To elucidate the mechanism by which *LOC101929709* might be involved in M1 infiltration, we identified the M1 infiltration-related genes that were also relevant to *LOC101929709*, including *PSMB8*, *PSMB9*, *FOSB*, *IFI27*, and *ISG15* using a cutoff of *r >0.4* or <−0.4 and P <*0.05* (**Figure 2F**). Based on the strong correlation of these genes, we considered that the commonly regulated genes, including lncRNAs, could be responsible for M1 infiltration in a synergistic manner, thus revealing the potential mechanisms of M1 infiltration (**Figure 2G**).

### Gene Modules Associated With Predicted pSS-Specific Immune Infiltration Patterns

**Figure 3:**
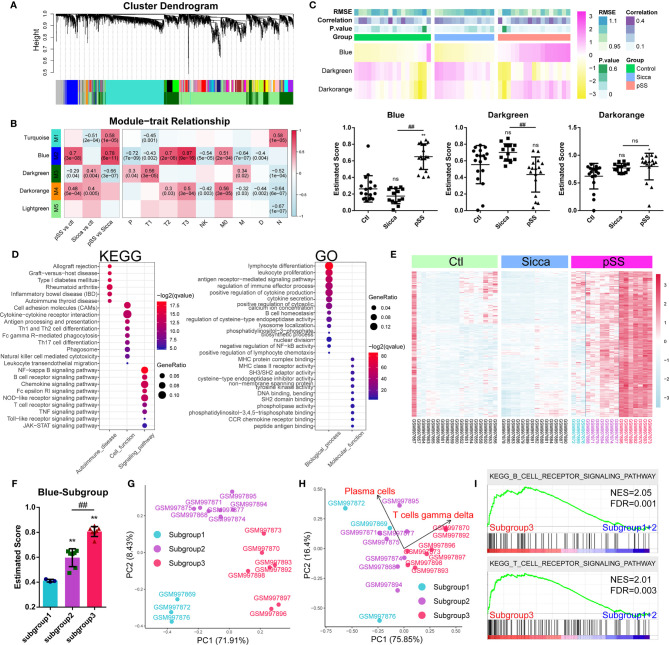
Gene modules associated with pSS-specific immune infiltration patterns. **(A)** Gene dendrogram calculated by average linkage hierarchical clustering. The color row underneath the dendrogram shows the assigned original module and the merged module. **(B)** Heatmap of the correlation between module eigengenes and disease or immune-infiltration features. The value in each square reflects the Pearson correlation coefficient, with the P value in parentheses. pSS *vs* ctl, comparison of pSS *versus* control; “Sicca” *vs* ctl, comparison of “Sicca” *versus* control; pSS *vs* “Sicca”, comparison of pSS *versus* “Sicca”. P, plasma cells; T1, CD8 T-cells; T2, memory activated CD4 T-cells; T3, gamma delta T-cells; NK, resting NK cells; M0, M0 macrophages; M, activated mast cells; D, resting dendritic cells; N, neutrophils. **(C)** Enriched score of the Blue, Darkgreen, and Darkorange immune-relevant modules in control, “Sicca”, and pSS samples estimated using the ssGSEA method. *P < 0.05, **P < 0.01, “Sicca” or pSS *versus* control; ##P < 0.01, pSS *versus* “Sicca”; all P values were estimated using one-way ANOVA. **(D)** Functional annotation of the Blue module using GO and KEGG. **(E)** Heatmap of gene expression of the Blue module in the control, “Sicca”, and pSS samples. Three subgroups were identified (color blue, purple, and red). **(F)** Enriched score of the Blue modules of the three subgroups in pSS. **P < 0.01, “Sicca” or pSS versus control; ^##^P < 0.01, pSS *versus* “Sicca”; P values were estimated using one-way ANOVA. **(G)** Principle component analysis (PCA) of the three subgroups of pSS samples based on the Blue module. **(H)** PCA of the three subgroups of pSS samples based on the disease-specific infiltrated immune cells. Plasma cells and gamma T-cells were the robustly-contributing elements in PCA. **(I)** Gene Set Enrichment Analysis (GSEA) of the subgroups of pSS. NES, normalized enrichment score; FDR, false discovery rate, P values were estimated using one-way ANOVA and adjusted with the Benjamini–Hochberg method. ns, not significant.

Following the identification of common genes potentially linked to M1 infiltration, we further investigated the mechanisms of pSS-specific immune infiltration patterns. We employed the *WGCNA* method, where we assigned coexpressed genes into modules and calculated the relationship of modules and phenotypes, including disease type and infiltration subpopulation, to identify disease- or subpopulation-relevant modules (**Figure 3A** and **Supplemental Table 7**). As shown in **Figure 3B**, we identified three significant modules, *Blue*, *Darkgreen*, and *Darkorange*, as pSS-relevant modules. Interestingly, the *Blue* module was shown to be not significant for “Sicca” but was highly relevant to multiple infiltrated subpopulations except for *neutrophils*, suggesting that this module might be linked to the pSS-specific immune infiltration patterns. Moreover, we applied the *ssGSEA* method to calculate the estimated score of these three modules in normal, “Sicca”, and pSS. We accordingly observed that the estimated score of the *Blue* module was consistently higher in pSS compared with that in “Sicca” and normal, whereas the estimated score of the *Darkgreen* module was not significant in either pSS or “Sicca” in comparison with that in normal (**Figure 3C**). The *Darkorange* module was demonstrated to be significant in the comparison between pSS and normal, but not significant for pSS *versus* “Sicca” (**Figure 3C**). Similarly, the results of ssGSEA suggested that the disease-specific difference of pSS should be attributed to the *Blue* and not to the other two modules. Taken together, the *Blue* module was the module selected to be subjected to further analysis.

We performed functional annotations of the *Blue* module that revealed the potential mechanisms of immune infiltration (**Figure 3D**). KEGG analysis suggested that the *Blue* module was relevant to multiple autoimmune diseases, such as rheumatoid arthritis, inflammatory bowel disease (IBD), and autoimmune thyroid disease. This module was also found to be involved in multiple immune-relevant signaling pathways, including the *NF-kappa B* signaling pathway, B-cell receptor signaling pathway, chemokine signaling pathway, and NOD-like receptor signaling pathway. GO analysis revealed that the genes of the *Blue* module were involved in the functions of lymphocytes and leukocytes, as well as in the production and secretion of cytokines through the regulation of the function of CCR receptors, MHC protein complexes, cysteine-type endopeptidases, and tyrosine kinases. These result suggested the functional relevance of the *Blue* module in predicted pSS-specific immune infiltration patterns.

Based on the *Blue* module, we identified three subgroups in the pSS samples, the high-, medium-, and low-scoring groups (**Figure 3C**).Consecutively, we plotted the gene expression pattern and calculated the estimated score of the subgroups (**Figures 3E, F**). We accordingly found that the three subgroups exhibited a correlation to this module. PCA results showed that the *Blue* module could serve as the parameter separating these three subgroups, whereas the immune-infiltration patterns of subgroups 1 and 2 showed no significant difference (**Figures 3G, H**), suggesting that the changes in the level of gene expression of the Blue module might occur before the remodeling of the infiltration pattern, hence driving the onset of this remodeling. However, subgroup 3 was shown to be significantly different from the other two in terms of both gene expression and infiltration pattern. To this end, we merged subgroups 1 and 2 into a single subgroup, namely subgroup A, with subgroup 3 being renamed as subgroup B. Further pathway analysis showed that the difference between subgroups A and B was the T- and B-cell receptor signaling pathways (**Figure 3I**).

### CTA-250D10.23 Associated With Predicted pSS-Specific Immune Infiltration Patterns by Chemokine Pathways

**Figure 4:**
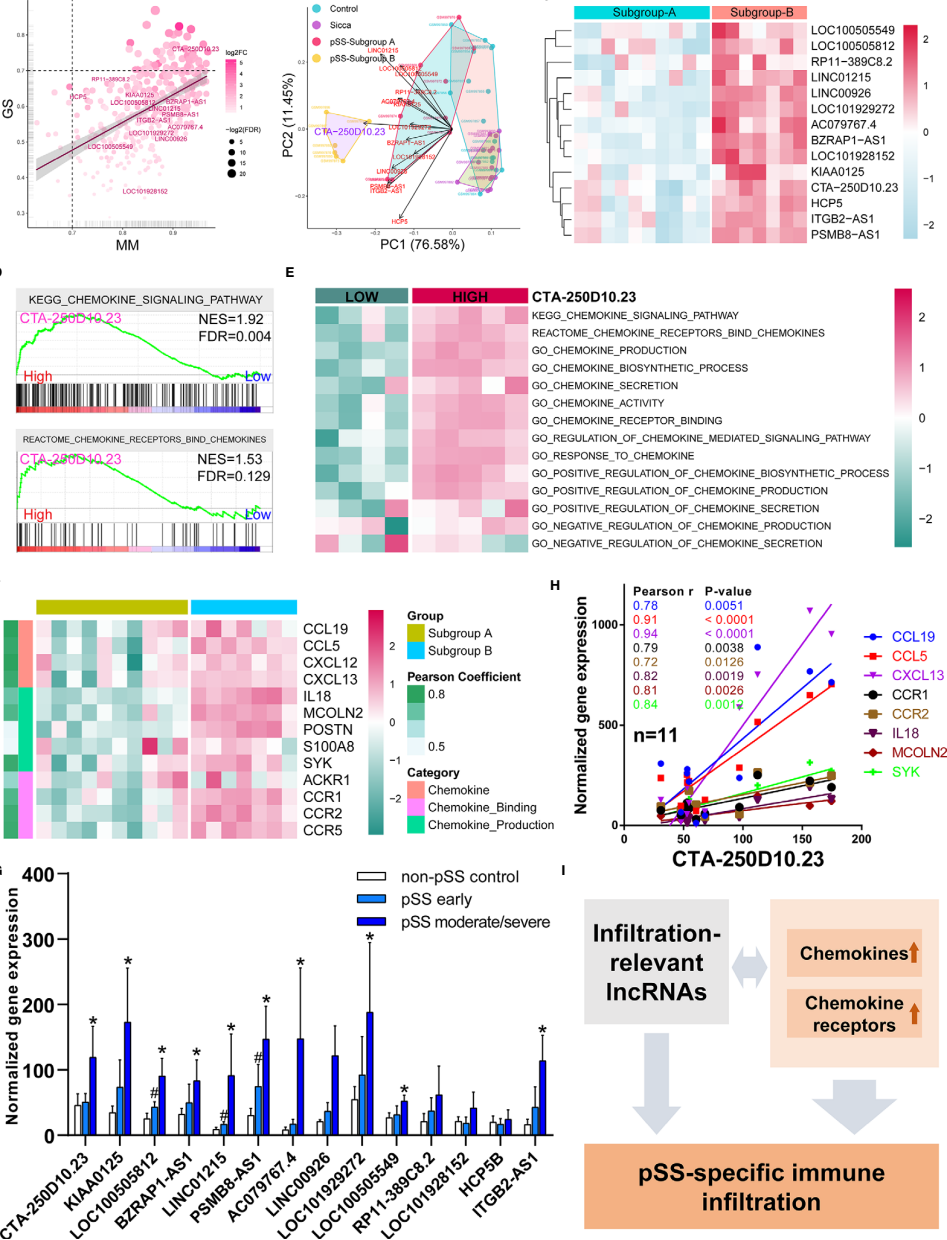
CTA-250D10.23 lncRNA associated with the predicted pSS-specific immune infiltration patterns and chemokine pathways. **(A)** Scatter plot of the gene significance (GS) for weight *vs* the module membership (MM) in the Blue module, estimated by WGNCA. Fourteen lncRNAs were identified in the Blue module, among which *CTA-250D10.23* was the most significant one based GS and MM. **(B)** PCA of control, “Sicca”, pSS subgroup A and pSS subgroup B based on the 14-lncRNA signature in the Blue module. The *CTA-250D10.23* lncRNA was the main contributor in the PCA plot in distinguishing between the pSS and non-pSS, and the subgroup A and subgroup B **(C)** Heatmap of the expression of 14 lncRNAs in the pSS subgroup A and B **(D)** GSEA of high- and low-expressing CTA-250D10.23 pSS samples shows the relevance to chemokine pathways. **(E)** ssGSEA estimated scores of chemokine pathways in high- and low-expressing *CTA-250D10.23* pSS samples. The estimated scores of high-expressing group were higher than the low-expressing group. **(F)** Heatmap of the expression of chemokine-relevant genes coexpressed with *CTA-250D10.23* lncRNA. **(G)** The expression of 14 lncRNAs in pSS patients in independent validation dataset GSE23117. 10 out of 14 lncRNAs were up-regulated in early or moderate/severe pSS or both. #P < 0.05, early pSS *versus* control; *P < 0.05, moderate/severe pSS *versus* control. **(H)** The correlation of the expression of *CTA-250D10.23* lncRNA and the genes involved in chemokine pathways in the pSS patients (n = 11) of the independent validation dataset GSE23117. *CTA-250D10.23* shows strong correlation with *CXCL13* and *CCL5*. **(I)** A model of potential mechanisms of the immune infiltration-relevant *CTA-250D10.23* lncRNA and chemokine pathways linked to predicted pSS-specific immune infiltration patterns.

Based on our finding that lncRNAs associated with the common phenotype of “Sicca” and pSS, we also investigated whether lncRNAs were involved in the differences in immune infiltration patterns. We identified 14 lncRNAs in the *Blue* module, including *CTA-250D10.23*, *RP11-389C8.2*, *KIAA0125*, *HCP5*, *LOC100505812*, *BZRAP1-AS1*, *LINC01215*, *PSMB8-AS1*, *ITGB2-AS1*, *AC079767.4*, *LINC00926*, *LOC100505549*, *LOC101928152*, and *LOC101929272*. According to gene significance (**Figure 4A**), *CTA-250D10.23* lncRNA was identified as the most significant gene contributing to this module among these 14 lncRNAs. Our PCA and heatmap plot results showed that the 14-lncRNA signature was able to separate the pSS samples from the normal and “Sicca” samples and further distinguish between the two subgroups of pSS (**Figures 4B, C**). Again, *CTA-250D10.23* was the most significant contributor in PCA, suggesting the critical connection between *CTA-250D10.23* and the predicted disease-specific immune infiltration pattern.

To better understand the potential mechanisms, we compared the differences observed in the pathways between the high- and low-expressing *CTA-250D10.23* groups. GSEA results showed that the genes involved in the chemokine signaling pathway were more potently up-regulated in the high- than in the low-expressing group (**Figure 4D**). ssGSEA results also showed the relevance of *CTA-250D10.23* lncRNA in multiple chemokine-relevant signaling pathways, including chemokine biosynthesis and activity, except for chemokine secretion (**Figure 4E**). Moreover, we identified the genes relevant to chemokine signaling pathways that were also coexpressed with *CTA-250D10.23* lncRNA. As shown in **Figure 4F**, these genes, such as CXCLs and CCRs, were up-regulated in subgroup B compared with those in subgroup A, suggesting the relevance of *CTA-250D10.23* lncRNA, chemokine, and immune infiltration. Finally, we employed an independent dataset to validate the expression of these 14 lncRNAs in pSS patients and correlations of *CTA-250D10.23* and the genes involved in chemokine pathway (**Figures 4G, H**). Strikingly, 10 out of 14 lncRNAs were up-regulated in early or moderate/severe pSS or both, including *CTA-250D10.23*. Besides, *CTA-250D10.23* showed strong correlation with *CXCL13* and *CCL5* in validation.

## Discussion

The pivotal point in distinguishing between pSS and “Sicca” is the definition of disease-specific features of immunopathological mechanisms. In the present study, we initially investigated the structural mode of disease-specific immune infiltration patterns among groups of normal donors, patients with pSS, and patients with non-pSS “Sicca” and found the specific structural changes of this pattern characterized by an sharply elevated immune proportion in pSS compared with that of the others. In addition, we identified the common genes, including lncRNAs, linked to these consistent immune-infiltration features, which might contribute to the mechanism of mucous membrane dryness. The most important finding was the identification of immune infiltration-relevant targets and the underlying mechanisms that might linked to pSS-specific immune infiltration patterns, in particular, the links between chemokine genes associated with predicted immune features and certain lncRNAs.

pSS is an autoimmune condition characterized by systemic B-cell activation and product of autoantibody within the salivary gland. Based on previous research associated with pSS, the immune-mediated inflammatory cellular infiltration has been associated with ectopic production of lymphoid chemokines, T/B cell segregation, and ectopic germinal center formation (21–23) as well as lymphocytic infiltration with a high degree of correlation between both morphological changes and immunological patterns and SS pathogenesis (24). Exocrine gland lymphocytic infiltration and B-cell hyperactivation have been recognized as the most relevant characteristics of the pSS disease model (25), resulting in abnormal and dysregulated responses from the activation of B-cells, such as the systemic elevation in immunoglobulins and autoantibodies (24, 26, 27).

Regarding the analysis of the 22 types of immune cell infiltration in this study, the pSS group demonstrated a more active ratio of immune response in six subpopulations, such as that of naïve B-cells. Glauzy et al. (28) also illustrated that the frequencies of mature naive B-cells expressing increased autoreactive antibodies in patients with pSS and the impairment of peripheral B-cell tolerance might be potentially correlated with pSS pathogenesis. Although we noted that a total of 10 subpopulations exhibited disease correlation with pSS, only four types of immune cells, including memory activated CD4 T-cells, CD8 T-cells, gamma delta T-cells, and plasma cells, were demonstrated to be involved in the distinction between the control, “Sicca”, and pSS. The progressive enlargement of pSS inflammatory foci with B-cell hyperactivity was shown to be a key factor related to the pathogenesis and progression of pSS, whereas T-cells and plasma cells were only found to contribute to the disease-specific immune infiltration of pSS, which differed from that of the control and “Sicca”.

Memory CD4+ T-cells are highly relevant to autoimmune diseases because of their long-lived nature, efficient responses to antigens, and specific potential to mediate recurring autoimmune responses (29). However, many critical questions about the potential contribution of memory CD4+ T-cells to autoimmune diseases remain unanswered. Joachims et al. (30) reported the crucial study that focused on the difference between pSS and non-SS “Sicca” in terms of memory CD4+ T-cells and revealed that the proportions and numbers of memory CD4+ T-cells in the salivary gland were related to key SS features; however, the interrelationship between memory T-cells and the specificity of pSS is still not fully understood. Determining the immunobiological contributions of CD4+ memory and CD8+ T-cells in chronic autoimmune diseases is pivotal toward developing improved targeted therapies for CD4+ or CD8+ T-cell-driven autoimmune diseases. In addition, the increased gamma delta T-cell population potentially contributes to distinguish pSS from “Sicca”. Ichikawa et al. reported that the proportion of activated cells in the gamma/delta + T-cell subset was significantly higher in the peripheral blood of the pSS patients than in the control and the frequency of activated cells was correlated with the duration of disease in pSS patients (31). Following their study, Gerli et al. reported that the relative elevated proportion of gamma/delta+ T-cell subset proposed that this T-cell subpopulation may be functional in the pathological immune response encountered in pSS (32). However, the solid evidence of the involvement of this T-cell subset is scarce so far. Recently some notable studies driven by the great interests of this subset have added a novel layer of understanding of its functions in multiple fields, including but not limited to tumor microenvironment and lung immune response. Generally, this T-cell subset has a role in tissue homeostasis and in surveillance of infection (33). In future study, the experimental validation and the demonstration of gamma delta T-cells in pSS would be of great interest because our understanding of its role in pSS pathogenesis is still rare.

When investigating the underlying mechanisms of the immune infiltration pattern, we found a notable involvement of lncRNAs. A pilot study by Dolcino et al. (34) identified 199 differentially expressed lnRNAs, such as *LINC00657* and *LINC00511*, in peripheral blood mononuclear cells (PBMCs), suggesting that lncRNAs might be involved in the pathogenesis of pSS. Inamo et al. (35) demonstrated that the *LINC00487* lncRNA was significantly up-regulated in B-cells and associated with the dysregulation of B-cells in pSS. In addition, the *PVT1* lncRNA was found to be involved in glycolytic metabolism reprogramming and proliferation upon activation of CD4+ T-cells (36). However, the function of lncRNAs remains elusive. In this study, we identified 14 lncRNAs associated with immune infiltration events, the functional annotation of which also highlighted their roles in immune-relevant signaling pathways, including the NF-kappa B signaling pathway, B-cell receptor signaling pathway, chemokine signaling pathway, and NOD-like receptor signaling pathway (**Figure 4I**). Given that these pathways are highly relevant to pSS pathogenesis, it would suggest a strong connection between these lncRNAs and disease. In particular, the *CTA-250D10.23* lncRNA, which was identified as the most significant gene associated with infiltration events, is located close to *TNFRSF13C* and annotated as lnc-TNFRSF13C-1 in the LNCipedia database (version 5.2), which might suggest the link between *CTA-250D10.23* and *TNFRSF13C* and the potential role of *CTA-250D10.23* in B-cell survival. Interestingly, our study found a strong correlation between *CTA-250D10.23* and CXCL13. Serum CXCL13 is a biomarker of salivary gland local pathology (37) and local increased of CXCL13 also serves as one of features of the pSS patient subset (38). As an echo to these lines of evidence, CXCL13 was identified as the most robustly up-regulated gene in pSS (**Supplemental Figure 1**). With a strong connection to this chemokine, it would not be surprising that, even if it is a speculation yet, *CTA-250D10.23* also has an incredible role in pSS by the involvement of CXCL13.

The previous study performed by Woon et al. found seven pSS-relevant co-expressed gene modules by WGCNA, of which four were up-regulated and three were downregulated in pSS patients compared with the non-pSS sicca and controls. In their study, they mainly distinguished pSS patients from the non-pSS sicca and controls, while they did not make the comparison of pSS and non-pSS sicca. The main goal of this study is to compare the pSS patients with the non-pSS sicca patients to explore the pSS-specific mechanisms, in particular, the immunopathogenesis and disease-specific immune infiltration pattern. As shown in **Supplemental Figure 3** and **Supplemental Table 8**, the immune infiltration-relevant Blue module identified in our study has a big overlap with the Magenta and the Brown module out of the seven pSS-relevant modules identified by Wong et al. (7), suggesting the consistency between the two studies. Beyond these consistent findings, we also identified 27 genes that were not mentioned in Wong’s results, which include 12 lncRNAs that we identified as the pSS immune infiltration-relevant lncRNAs in our study.

Several limitations in the present study should be acknowledged. First, although the 14 lncRNAs were validated by another independent dataset, all data analyses were based on a single dataset, which might downgrade the universality of the conclusion. Secondly, limitations of data analysis study should be taken into consideration and the interpretations and conclusions should be addressed carefully. It is no doubt that the evidence from clinical human samples would be the most expected way to validate the predicted immune infiltration and add another layer of significance to this study. Moreover, further studies of pSS-specific animal models may also help to confirm our results. Except for the limitations of *in-silico* study, the adjustment for potential confounding clinical features would be helpful to get more reliable results and conclusions. However, from the database we did not find the clinical information corresponding to each gene profile, which means it would be hard to eliminate the bias induced by the potential confounding clinical features.

## Conclusion

Aberrant predicted disease-specific immune infiltration patterns and relevant genes revealed the potential immunopathogenesis in pSS and provided some clues for the improved immunotherapy of the disease by targeting specific immune cells and genes.

## Data Availability Statement

The original contributions presented in the study are included in the article/**Supplementary Material**. Further inquiries can be directed to the corresponding authors.

## Author Contributions

JiZ, study design and quality control. CC, literature and dataset search. JiZ and JuZ, data analysis. JW, CC, and JuZ, data verification. RC, YS, and RT, data interpretation. JiZ and CC, figure adaptation. JiZ, JuZ, and JW, drafting and final approval. JuZ and JW, funding support. All authors contributed to the article and approved the submitted version.

## Funding

This study was supported by the National Natural Science Foundation of China (Grant Nos. 81771114 and 81970967) and Grants-in-Aid for Research Activity Start-up (19K24154) from the Japan Society for the Promotion of Science.

## Conflict of Interest

The authors declare that the research was conducted in the absence of any commercial or financial relationships that could be construed as a potential conflict of interest.

## Supplementary Material

Supplementary Figure 1 The volcano plot of differentially-expressed genes in the comparison of pSS or “Sicca” versus control.

Supplementary Figure 2 The overlapped genes of the 32 commonly regulated genes and LM22 M1 prediction reference.

Supplementary Figure 3 The genes in the Blue module overlapped with the Magenta and the Brown modules identified in the original publication.

Supplementary Table 1 The LM22 reference employed in immune infiltration estimation.

Supplementary Table 2 The predicted relative abundance of 22 types of immune cell estimated by CIRBERSORT.

Supplementary Table 3 The comparison of immune infiltration abundance of pSS or “Sicca” *versus* control.

Supplementary Table 4 The differential expression analysis of gene expression of pSS *versus* control.

Supplementary Table 5 The differential expression analysis of gene expression of “Sicca” *versus* control.

Supplementary Table 6 Immune score signature employed in the estimation of immune state of control, pSS and “Sicca”.

Supplementary Table 7 The gene modules and genes identified by WGCNA.

Supplementary Table 8 The overlapped genes identified by the comparison with the original publication.

## References

[1] Vivino FB. Sjogren’s syndrome: Clinical aspects. Clin Immunol (2017) 182:48–54. 10.1016/j.clim.2017.04.005 28428095

[2] Ramos-Casals M Brito-Zeron P Siso-Almirall A Bosch X. Primary Sjogren syndrome. Bmj (2012) 344:e3821. 10.1136/bmj.e3821 22700787

[3] Mariette X Criswell LA. Primary Sjogren’s Syndrome. New Engl J Med (2018) 378(10):931–9. 10.1056/NEJMcp1702514 29514034

[4] Stapleton F Alves M Bunya VY Jalbert I Lekhanont K Malet F. TFOS DEWS II Epidemiology Report. Ocular Surface (2017) 15(3):334–65. 10.1016/j.jtos.2017.05.003 28736337

[5] Sjogren H. On knowledge of the keratoconjunctivitis sicca. VII. The sicca syndrome–an autoimmune disease. Acta Ophthalmol (1968) 46(2):201–6. 10.1111/j.1755-3768.1968.tb05177.x 5755677

[6] Singh PB Young A Homayouni A. Distorted Taste and Impaired Oral Health in Patients with Sicca Complaints. Nutrients (2019) 11(2). 10.3390/nu11020264 PMC6412562 30682880

[7] Horvath S Nazmul-Hossain AN Pollard RP Kroese FG Vissink A Kallenberg CG. Systems analysis of primary Sjogren’s syndrome pathogenesis in salivary glands identifies shared pathways in human and a mouse model. Arthritis Res Ther (2012) 14(6):R238. 10.1186/ar4081 23116360 PMC3674589

[8] Baer AN Walitt B. Update on Sjogren Syndrome and Other Causes of Sicca in Older Adults. Rheumatic Dis Clinics North America (2018) 44(3):419–36. 10.1016/j.rdc.2018.03.002 PMC6245643 30001784

[9] Patel R Shahane A. The epidemiology of Sjogren’s syndrome. Clin Epidemiol (2014) 6:247–55. 10.2147/CLEP.S47399 PMC4122257 25114590

[10] Brito-Zeron P Baldini C Bootsma H Bowman SJ Jonsson R Mariette X. Sjogren syndrome. Nat Rev Dis Primers (2016) 2:16047. 10.1038/nrdp.2016.47 27383445

[11] Moutsopoulos HM. Sjogren’s syndrome: autoimmune epithelitis. Clin Immunol Immunopathol (1994) 72(2):162–5. 10.1006/clin.1994.1123 8050187

[12] Hillen MR Ververs FA Kruize AA Van Roon JA. Dendritic cells, T-cells and epithelial cells: a crucial interplay in immunopathology of primary Sjogren’s syndrome. Expert Rev Clin Immunol (2014) 10(4):521–31. 10.1586/1744666X.2014.878650 24450381

[13] Ritchie ME Phipson B Wu D Hu Y Law CW Shi W. limma powers differential expression analyses for RNA-sequencing and microarray studies. Nucleic Acids Res (2015) 43(7):e47. 10.1093/nar/gkv007 25605792 PMC4402510

[14] Newman AM Liu CL Green MR Gentles AJ Feng W Xu Y. Robust enumeration of cell subsets from tissue expression profiles. Nat Methods (2015) 12(5):453–7. 10.1038/nmeth.3337 PMC4739640 25822800

[15] Yoshihara K Shahmoradgoli M Martinez E Vegesna R Kim H Torres-Garcia W. Inferring tumour purity and stromal and immune cell admixture from expression data. Nat Commun (2013) 4:2612. 10.1038/ncomms3612 24113773 PMC3826632

[16] Hanzelmann S Castelo R Guinney J. GSVA: gene set variation analysis for microarray and RNA-seq data. BMC Bioinf (2013) 14:7. 10.1186/1471-2105-14-7 PMC3618321 23323831

[17] Langfelder P Horvath S. WGCNA: an R package for weighted correlation network analysis. BMC Bioinf (2008) 9:559. 10.1186/1471-2105-9-559 PMC2631488 19114008

[18] Yu G Wang LG Han Y He QY. clusterProfiler: an R package for comparing biological themes among gene clusters. Omics A J Integr Biol (2012) 16(5):284–7. 10.1089/omi.2011.0118 PMC3339379 22455463

[19] Subramanian A Tamayo P Mootha VK Mukherjee S Ebert BL Gillette MA. Gene set enrichment analysis: a knowledge-based approach for interpreting genome-wide expression profiles. Proc Natl Acad Sci USA (2005) 102(43):15545–50. 10.1073/pnas.0506580102 PMC1239896 16199517

[20] Greenwell-Wild T Moutsopoulos NM Gliozzi M Kapsogeorgou E Rangel Z Munson PJ. Chitinases in the salivary glands and circulation of patients with Sjogren’s syndrome: macrophage harbingers of disease severity. Arthritis Rheumatism (2011) 63(10):3103–15. 10.1002/art.30465 PMC3183169 21618203

[21] Barone F Bombardieri M Manzo A Blades MC Morgan PR Challacombe SJ. Association of CXCL13 and CCL21 expression with the progressive organization of lymphoid-like structures in Sjogren’s syndrome. Arthritis Rheumatism (2005) 52(6):1773–84. 10.1002/art.21062 15934082

[22] Salomonsson S Jonsson MV Skarstein K Brokstad KA Hjelmstrom P Wahren-Herlenius M. Cellular basis of ectopic germinal center formation and autoantibody production in the target organ of patients with Sjogren’s syndrome. Arthritis Rheumatism (2003) 48(11):3187–201. 10.1002/art.11311 14613282

[23] Saraux A Pers JO Devauchelle-Pensec V. Treatment of primary Sjogren syndrome. Nat Rev Rheumatol (2016) 12(8):456–71. 10.1038/nrrheum.2016.100 27411907

[24] Dinescu SC ForTofoiu MC Bumbea AM Ciurea PL Busuioc CJ Musetescu AE. Histopathological and immunohistochemical profile in primary Sjogren’s syndrome. Romanian J Morphol Embryol Rev Roumaine Morphol Embryol (2017) 58(2):409–17. 28730224

[25] Mariette X. Lymphomas complicating Sjogren’s syndrome and hepatitis C virus infection may share a common pathogenesis: chronic stimulation of rheumatoid factor B cells. Ann Rheumatic Dis (2001) 60(11):1007–10. 10.1136/ard.60.11.1007 PMC1753426 11602464

[26] Tincani A Andreoli L Cavazzana I Doria A Favero M Fenini MG. Novel aspects of Sjogren’s syndrome in 2012. BMC Med (2013) 11:93. 10.1186/1741-7015-11-93 23556533 PMC3616867

[27] Salomonsson S Wahren-Herlenius M. Local production of Ro/SSA and La/SSB autoantibodies in the target organ coincides with high levels of circulating antibodies in sera of patients with Sjogren’s syndrome. Scandinavian J Rheumatol (2003) 32(2):79–82. 10.1080/03009740310000076 12737325

[28] Glauzy S Sng J Bannock JM Gottenberg JE Korganow AS Cacoub P. Defective Early B Cell Tolerance Checkpoints in Sjogren’s Syndrome Patients. Arthritis Rheumatol (2017) 69(11):2203–8. 10.1002/art.40215 PMC6062007 28704602

[29] Raphael I Joern RR Forsthuber TG. Memory CD4(+) T Cells in Immunity and Autoimmune Diseases. Cells (2020) 9(3):531. 10.3390/cells9030531 PMC7140455 32106536

[30] Joachims ML Leehan KM Dozmorov MG Georgescu C Pan Z Lawrence C. Sjogren’s Syndrome Minor Salivary Gland CD4(+) Memory T Cells Associate with Glandular Disease Features and have a Germinal Center T Follicular Helper Transcriptional Profile. J Clin Med (2020) 9(7). 10.3390/jcm9072164 PMC7408878 32650575

[31] Ichikawa Y Shimizu H Yoshida M Takaya M Arimori S. T cells bearing gamma/delta T cell receptor and their expression of activation antigen in peripheral blood from patients with Sjogren’s syndrome. Clin Exp Rheumatol (1991) 9(6):603–9. 1722441

[32] Gerli R Agea E Muscat C Georgescu C Pan Z Lawrence C. Functional characterization of T cells bearing the gamma/delta T-cell receptor in patients with primary Sjogren’s syndrome. Clin Exp Rheumatol (1993) 11(3):295–9. 8353984

[33] Ribot JC Lopes N Silva-Santos B. gammadelta T cells in tissue physiology and surveillance. Nat Rev Immunol (2020). 10.1038/s41577-020-00452-4 33057185

[34] Dolcino M Tinazzi E Vitali C Del Papa N Puccetti A Lunardi C. Long Non-Coding RNAs Modulate Sjogren’s Syndrome Associated Gene Expression and Are Involved in the Pathogenesis of the Disease. J Clin Med (2019) 8(9):1349. 10.3390/jcm8091349 PMC6780488 31480511

[35] Inamo J Suzuki K Takeshita M Kassai Y Takiguchi M Kurisu R. Identification of novel genes associated with dysregulation of B cells in patients with primary Sjogren’s syndrome. Arthritis Res Ther (2020) 22(1):153. 10.1186/s13075-020-02248-2 32571405 PMC7310138

[36] Fu J Shi H Wang B Zhan T Shao Y Ye L. LncRNA PVT1 links Myc to glycolytic metabolism upon CD4(+) T cell activation and Sjogren’s syndrome-like autoimmune response. J Autoimmun (2020) 107:102358. 10.1016/j.jaut.2019.102358 31757716

[37] Colafrancesco S Priori R Smith CG Minniti A Iannizzotto V Pipi E. CXCL13 as biomarker for histological involvement in Sjogren’s syndrome. Rheumatology (2020) 59(1):165–70. 10.1093/rheumatology/kez255 31274159

[38] James JA Guthridge JM Chen H Lu R Bourn RL Bean K. Unique Sjogren’s syndrome patient subsets defined by molecular features. Rheumatology (2020) 59(4):860–8. 10.1093/rheumatology/kez335 PMC7188221 31497844

