# Supplementary figures and images for: Predicted Disease-Specific Immune Infiltration Patterns Decode the Potential Mechanisms of Long Non-Coding RNAs in Primary Sjogren’s Syndrome
Source: Front Immunol. 2021 Apr 14;12:624614. doi: 10.3389/fimmu.2021.624614 (PMC8079748; doi:10.3389/fimmu.2021.624614)

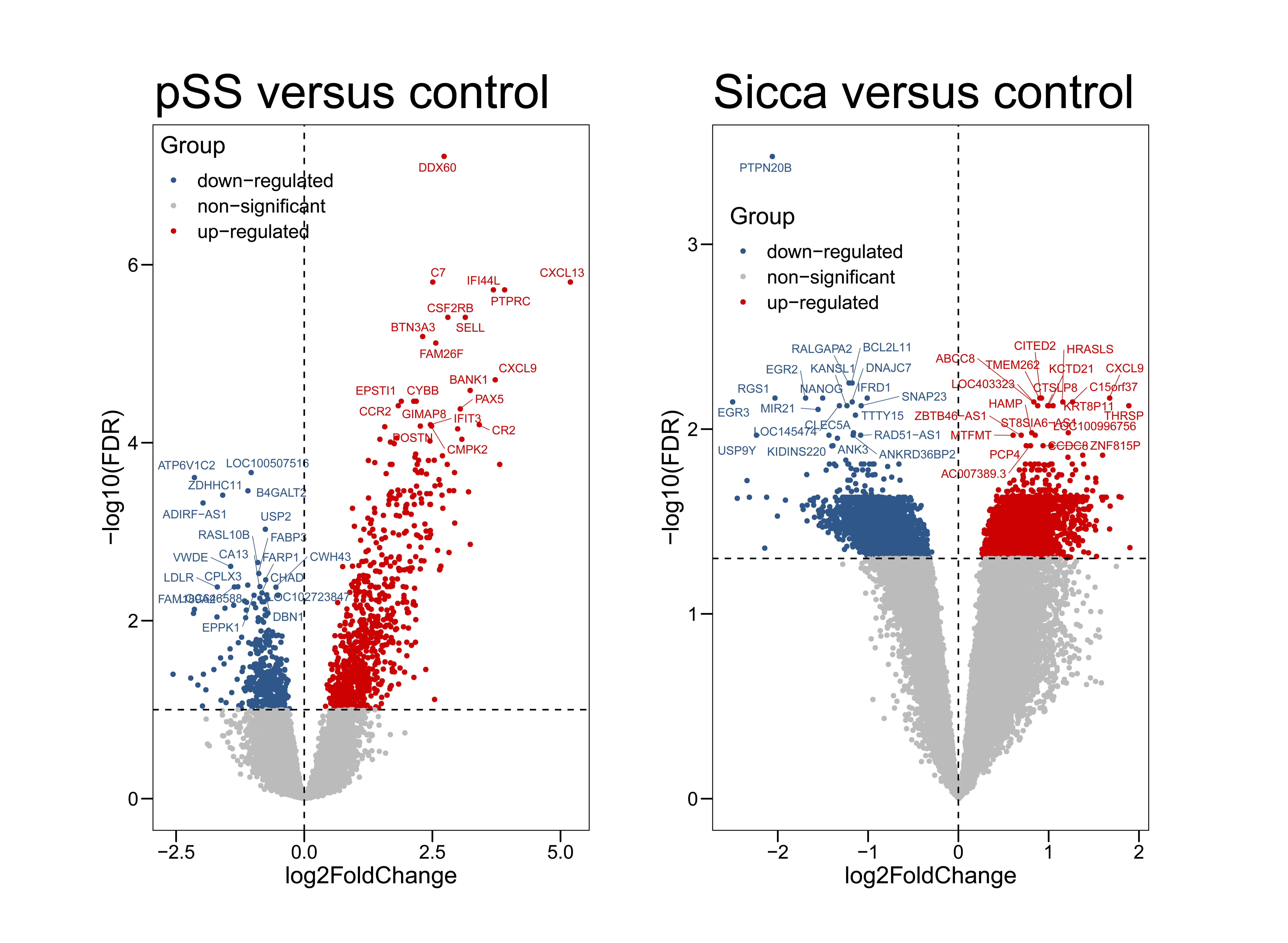

Supplement: Supplementary Figure 1 — The volcano plot of differentially-expressed genes in the comparison of pSS or “Sicca” versus control. [file Image_1.tif]

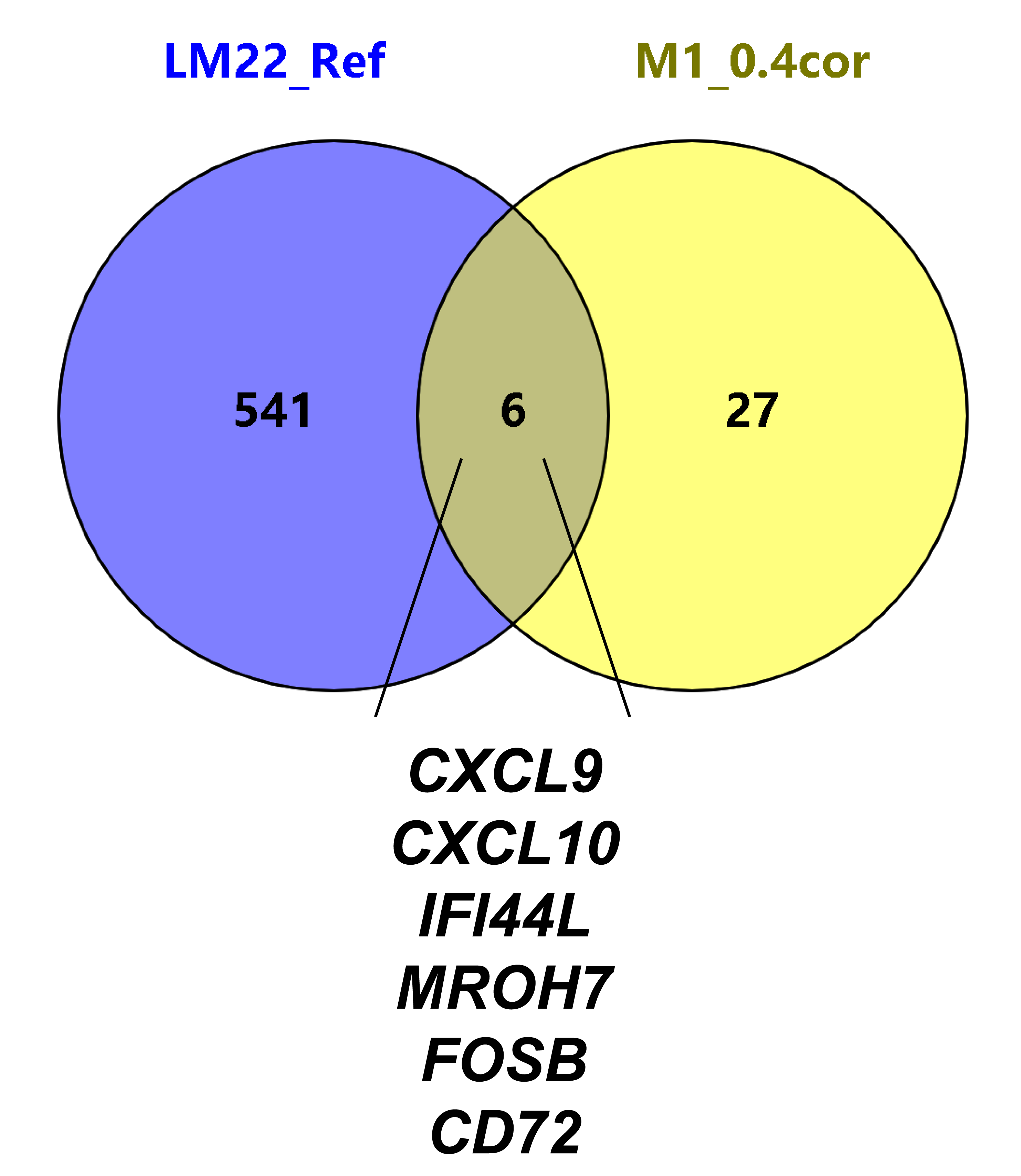

Supplement: Supplementary Figure 2 — The overlapped genes of the 32 commonly regulated genes and LM22 M1 prediction reference. [file Image_2.tif]

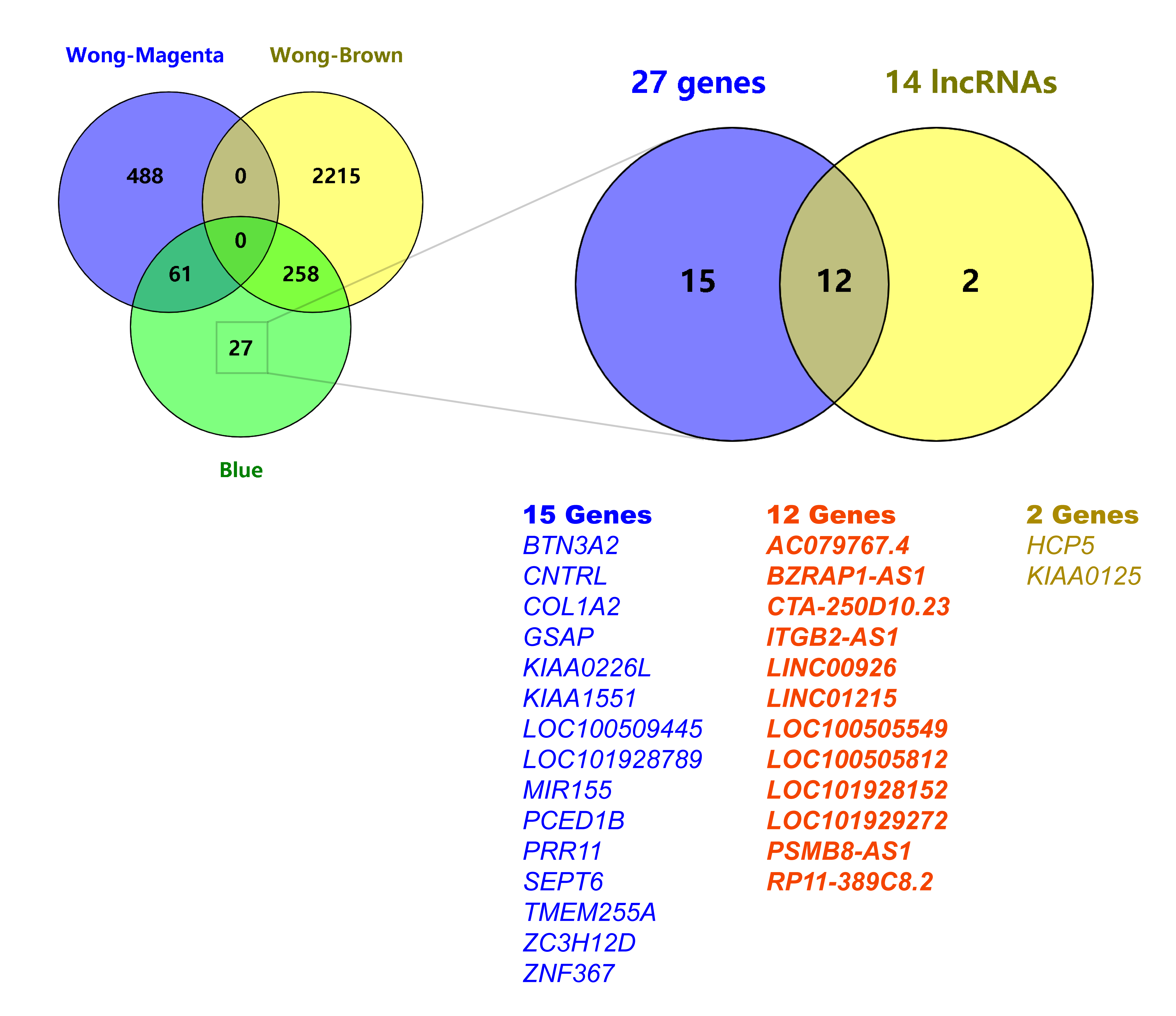

Supplement: Supplementary Figure 3 — The genes in the Blue module overlapped with the Magenta and the Brown modules identified in the original publication. [file Image_3.tif]
